# Short circuit: Transcription factor addiction as a growing vulnerability in cancer^☆^

**DOI:** 10.1016/j.sbi.2024.102948

**Published:** 2024-12

**Authors:** Molly Davies, Maeve Boyce, Eric Conway

**Affiliations:** School of Biomolecular and Biomedical Sciences, Conway Institute, University College Dublin, Dublin 4, Ireland

**Keywords:** Transcription factor, Core regulatory circuitry, oligoTRAFTAC, o'PROTAC, CRISPR screen, Cancer dependency, Transcription factor network, Cancer, Targeted therapy, Personalised medicine, Epigenetics

## Abstract

Core regulatory circuitry refers to the network of lineage-specific transcription factors regulating expression of both their own coding genes, and that of other transcription factors. Such autoregulatory feedback loops coordinate the transcriptome and epigenome during development and cell fate decisions. This circuitry is hijacked during oncogenesis resulting in cancer cell fate being maintained by lineage-specific transcription factors. Major advances in functional genomics and chemical biology are paving the way for a new generation of cancer therapeutics aimed at disrupting this circuitry through both direct and indirect means. Here we review these critical advances in mechanistic understanding of transcription factor addiction in cancer and how the advent of proteolysis targeting chimeras and CRISPR screen assays are leading the way for a new paradigm in targeted cancer treatments.

## Highlights

• Cancer cells rewire core regulatory circuitry of lineage-specific transcription factors to drive oncogenic gene expression.
• CRISPR screens have revealed lineage-specific transcription factors as exquisitely selective cancer dependencies.
• Chemical biology advances, such as proteolysis targeting chimeras, provide new avenues to target transcription factors.
• These advances could generate modular tools to customise cancer treatments based on transcription factor dependencies.

## Introduction

Transcription factors (TFs) are DNA-binding proteins that engage regulatory regions of DNA in a sequence-specific manner. They have critical roles in controlling many biological processes and signalling pathways by coordinating transcription and the epigenome [1,2]. The ability of TFs to bind to sequence-specific regions (motifs) of DNA is pivotal for directing their function to regulatory elements. This allows transcriptional machinery to be recruited to distinct sets of target loci to control transcriptional pathways or sets of genes. This recruitment was historically thought to result in the formation of stable, chromatin-bound complexes of TFs and transcriptional machinery. However, it was recently demonstrated that nucleosome eviction and deposition occur in dynamic, synergistic cycles coordinated by cooperative yet transient binding of TFs, chromatin remodelers, and RNA polymerase [3]. Furthermore, recent advances in tracking single molecule dynamics have revealed that TFs exhibit context-specific motif searching and binding dynamics, with varying residence times that contribute to cell-type specific transcriptional programs [4].

There are several classes of TFs that vary in their expression patterns, mechanism of action and role in organising chromatin accessibility and transcription. These include subtypes such as general, hormone receptor, and lineage-specific TFs.

General TFs are essential for the expression of all genes [5,6]. They are a key component of the preinitiation complex along with RNA polymerase II (Pol II). Pol II cannot bind to promoters in the absence of general TFs, and the establishment of the preinitiation complex recruits other key regulators to initiate transcription [5,6]. Hormone receptor TFs gain transcriptional activity in response to hormones that influence their nuclear localisation. Some hormone receptor TFs, such as the estrogen receptor (ER) and androgen receptor (AR), undergo conformational changes upon ligand binding which enable sequence recognition by exposing their DNA-binding domain [7,8]. Hormones involved in cell signalling pathways can modulate the transcriptional function of other hormone receptor TFs, including the NF-kB and STAT proteins which gain transcriptional activity in response to cytokines and other growth factors [9,10]. While many hormone receptors have a prominent role in driving cancer, in particular ER and AR, they have been extensively reviewed elsewhere [11].

The major focus of this review are lineage-specific TFs which control cell fate decisions and differentiation. Expression of such TFs is highly cell-type specific whereas general TFs are broadly expressed across all cell types. A subset of lineage-specific TFs have pioneering activity, including SOX2 [12], PAX7 [13], and FOXA1 [14,15], meaning they are important initiators of chromatin opening. Pioneer TFs are capable of binding their target sequences on nucleosomal DNA, which has been shown to weaken the interaction between DNA and the histone octamer, initiating nucleosome shuffling and chromatin remodelling events [16, 17, 18]. Importantly, it has been shown that the pioneering capability of a TF is highly context-dependent, with variables such as motif position on the nucleosome and epigenetic modifications impacting TF access [19,20]. As such, pioneer TFs are not defined as a strict class of TFs. Instead, the pioneering action of TFs, coupled with genome accessibility, can be viewed as a spectrum of “chromatin sensitivity”, dependent on cellular context and TF expression [19].

Nonpioneering lineage-specific TFs lack the ability to remodel chromatin directly, but instead bind to nucleosome-free regulatory elements to establish transcriptional networks which govern the expression of cell fate genes [21]. This transcriptional signalling generates a feedback network termed the ‘core regulatory circuitry’. The function of core regulatory TFs is perturbed during oncogenesis to promote a transcriptional network responsible for the maintenance of a nondifferentiated, pro-proliferative, tumourigenic cell state [22, 23, 24]. In general, this can be broadly viewed as a block in differentiation. Major advances in our understanding of the mechanism of, and technical capacity to map, core regulatory circuitry is yielding new attractive drug targets and means to develop tailored cancer treatments. Importantly, the lineage-specific expression patterns of these TFs provide an intriguing opportunity to exploit targeting these TFs for therapeutic benefit with minimal off-target consequences. Here we review these functional genomics and chemical biology advances and speculate on the future of the field in the coming years.

## Rewiring of core regulatory circuitry in cancer

**Figure 1:**
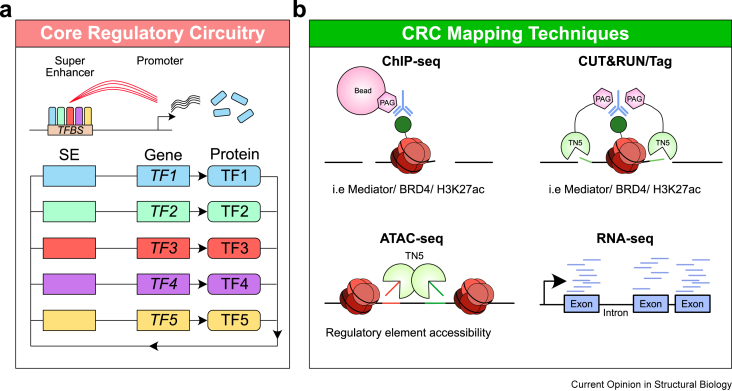
**Core regulatory circuitry is coordinated by lineage-specific transcription factors**. **a)** Core regulatory circuitry network showing autoregulatory positive feedback loops from sets of lineage-specific transcription factors. This circuitry is coordinated by transcription factors that control their own expression levels by binding to their promoters and superenhancers. This creates a positive feedback loop driving key gene expression programs. **b)** Epigenomic and transcriptomic methods that can be used to map core regulatory circuitry in a given cell type. Abbreviations: CRC - Core regulatory circuitry; SE - Superenhancer; TF- Transcription factor; TFBS - Transcription factor binding site.

The idea of core regulatory circuits (CRCs) driven by TFs is a well-established concept [25,26]. These CRCs consist of a set of key lineage-specific TFs that drive critical tissue-specific and cell type-specific gene expression programs. Crucially, this set of TFs can also regulate their own expression through binding to superenhancers and promoters that control expression of the lineage-specific TFs themselves in addition to their downstream targets [21,26] (Figure 1a).

CRCs have a critical role in the maintenance of cell fate and differentiation during normal tissue homeostasis and development. During oncogenesis, lineage-specific TFs hijack control of gene expression programs and the CRC to promote a cell state with the capacity to self-renew or block differentiation [22,27]. In this way, cancer cells become reliant on, or “addicted” to these altered transcriptional programs [23]. Moreover, the aberrant establishment and activation of superenhancers at key cell identity genes is a central part of this mechanism of TF addiction. While TFs themselves are frequently the subject of structural variants, fusions and mutations that can drive cancer [28], here we focus on wild-type TFs and how their function is repurposed leading to TF addiction in cancer.

Lineage-specific TFs have been intensely studied due to their importance in controlling CRCs [25]. Many different methods are available to map CRCs, including ChIP-seq/CUT&Tag of active enhancer related proteins (MED1 and BRD4) or histone modifications (H3K27ac), ATAC-seq, and RNA-seq [21,26,29,30] (Figure 1b). Results of such functional epigenomic experiments has fed databases allowing for the bioinformatics-based inference of CRCs and the prediction of core regulatory TFs governing the inferred networks which may function as genetic dependencies. This includes the CRCmapper [26] and Coltron [31] algorithms which identify superenhancer regions from H3K27ac ChIP-seq and scan superenhancer valleys for TF binding motifs to predict CRCs.

These CRC mapping methods, along with pharmacological and genomic studies, have identified critical roles for MYOD, IRF8, FOXA1, and SOX11 TFs in rhabdomyosarcoma, acute myeloid leukaemia (AML), prostate cancer, and neuroblastoma [22,27,32,33], respectively. Each of these TFs control their own expression and that of other lineage-specific TFs through superenhancer and promoter binding. In their absence, these circuits break down and cells can be induced to alternate fates including differentiation, decreased proliferation, or induction of apoptosis. For example, the IRF8-driven CRC blocks myeloid differentiation in AML but not control cells [32]. Contrastingly, PAX8 occupies *de novo* binding sites in ovarian cancer cells relative to healthy tissue [34]. Together, these show that rewiring may be a static accumulation of undifferentiated progenitors or a gain in *de novo* pathogenic CRCs in different contexts. This remains an outstanding question in the field.

Importantly, the application of these CRC mapping technologies on a single-cell and spatial level can allow high-resolution mapping of CRCs within specific cell populations [35]. Exemplary work examining these CRCs at the single-cell level has shown that TFAP2C and its regulatory network are critical in driving chemoresistance in breast cancer patients [35]. This advance has the potential to aid our understanding of which TFs have a major contribution in specific cell types, and this may be critical to understand the biology of mixed or rare cell populations in cancer, including treatment-resistant cells. Indeed, these techniques could be combined with clonal/cell genotyping techniques in a cancer context to determine whether cells with specific genotypes are differentially reliant on alternate lineage-specific TFs [36].

## Lineage-specific transcription factors are exquisitely specific genetic dependencies in cancer

Genetic experiments have identified key TF-cancer relationships that underlie the rewired CRCs in cancer cells. This includes critical roles in CRC regulation for TFs such as MYOD in rhabdomyosarcoma, AR in prostate cancer, and IRF8 in AML [22,32,33]. These individual studies have shed light on the importance of these lineage-specific TFs in the contexts of cancers related to that lineage. In these contexts, the lineage-specific TFs block cell fate decisions. Subsequently their loss facilitates differentiation down the appropriate lineage. This approach and phenotype would be highly desirable if translatable to a clinical setting.

Large-scale functional genomics efforts using pooled genome-wide RNAi and CRISPR libraries have facilitated the expansion of these observations to nearly all cancer types. Such screening methods have generated significant insights into cancer biology and synthetic lethal relationships over the last decade. The DepMap portal, which is fed by various projects managed by the Broad Institute, has the overarching aim of defining a “Cancer Dependency Map” [37, 38, 39, 40]. ‘Dependency scores’ which indicate a gene's essentiality for cancer cell line proliferation across hundreds of cancer cell lines are publicly available on DepMap. These data have facilitated the identification of key genotype–phenotype relationships between the survival of cancer cells and specific genes [41].

**Figure 2:**
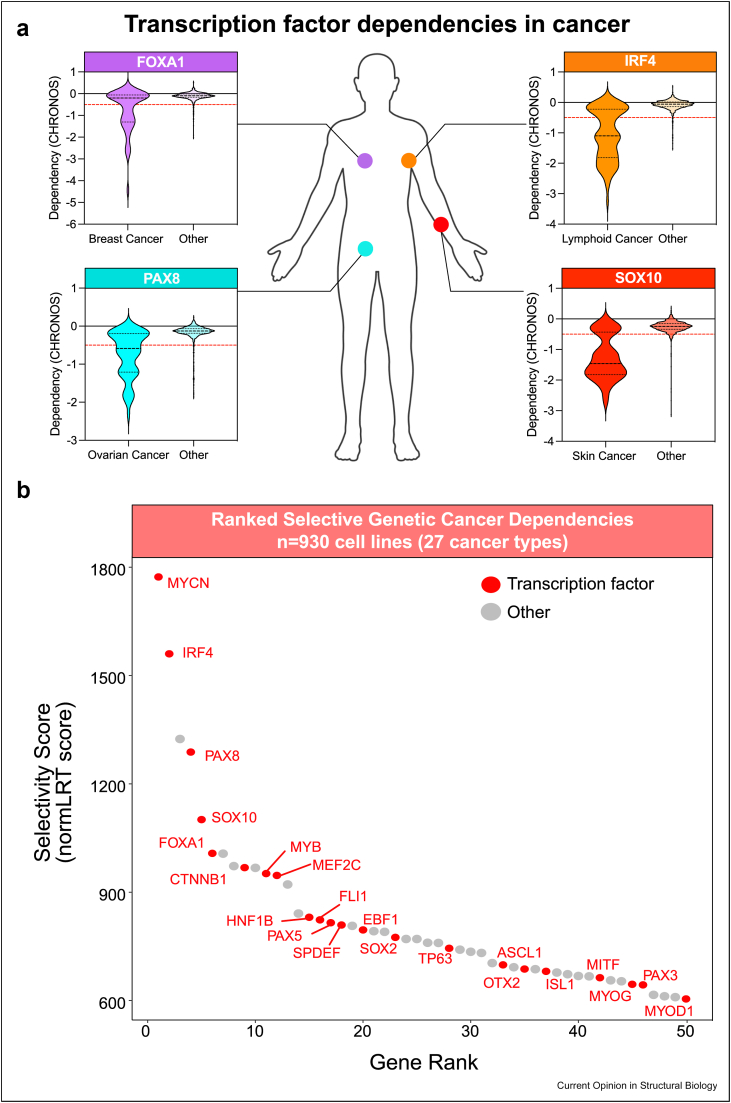
**Lineage-specific transcription factors exhibit exquisite specificity of dependency in specific cancer types**. **a)** Examples of highly specific genetic dependencies for ovarian, breast, lymphoid, and skin cancer subtypes on lineage-specific transcription factors PAX8, FOXA1, IRF4 and SOX10, respectively. Data generated by Ref. [40, 42]. Dataset used ‘CRISPR (DepMap Public 23Q2+Score Chronos)’. **b)** Dependency selectivity analysis on genome-wide CRISPR screen data from 930 cell lines across 27 cancer types in DepMap. Genes are ranked in order of selectivity (normLRT values). Transcription factors are highlighted in red. Adapted from Ref. [42].

One of the prominent results arising from these screening efforts is that the dependency of distinct cancer types on lineage-specific TFs is a common feature across the majority of cancers [42]. This includes FOXA1, IRF4, PAX8, and SOX10 which are respectively highly selectively essential in neuroblastoma, lymphoma, ovarian cancer, and glioma compared to all other cancer types (Figure 2a) [42]. In a recent study, a pan-cancer analysis of genome-wide CRISPR screen data was performed on 930 cancer cell lines across 27 cancer types to assess the selectivity of genetic dependencies to certain lineage or cancer types. Genes were ranked based on their ‘normal likelihood ratio test’ (normLRT) score, which assessed the deviation of dependency score (CERES) from the normal distribution as a measure of selective essentiality [42]. This was used as a means of determining the most selectively essential genes across all cancer types. Of the top 50 most selective ranked cancer dependency genes, 22 (44 %) encoded for TFs (Figure 2b), including 4 out of the top 5. These highly selective dependency TFs represented a broad range of cancer lineages including neuroblastoma, myeloid, lymphoid, sarcoma, breast, and more. This is a clear indication of the general importance of TFs in controlling CRCs in cancer cell fate and highlights their potential as specific therapeutic targets in cancer to block the oncogenic CRC.

There have been major advances in the development of cancer therapeutics that target epigenetic and chromatin regulators over the last 15 years. HDAC, EZH2, and DNMT inhibitors are prominent examples of FDA-approved treatments for different malignancies [43]. However, one drawback of approaches that target broadly expressed chromatin regulators is that they can have potentially severe side effects in tissues not related to the target tissue of interest due to the critical role of these regulators in maintaining adult stem cell homeostasis in different tissue compartments [44]. Accordingly, some of these epigenetic regulators that are being targeted chemically in clinical trials, including BRD4, are classed as ‘common essential’ dependencies in the DepMap database as they are essential for all cancer cell lines to survive [45]. This may suggest they are also potentially critical for normal tissue regeneration and homeostasis.

While lineage-specific TFs also have important roles in adult tissues these are significantly more limited due to their highly specific gene expression patterns. Therefore, approaches aiming to therapeutically target TFs would be a highly desirable avenue to implement treatments with minimal potential side effects and potent ‘on-target’ activity for the desired cancer cell type.

Taken together, the deregulation of transcription mediated by TFs as a major cancer driver presents TFs as promising putative targets. Unfortunately, TFs are notoriously difficult to target therapeutically, due to their intrinsically disordered nature and a lack of druggable domains within their secondary structure [46].

## Pharmacological targeting of transcription factor activity

Due to these biochemical limitations hindering typical efforts to drug TFs, novel approaches have been developed to inhibit TFs through both direct and indirect means. A common preclinical approach is to instead target chromatin regulators acting in complex with, or through the same pathways as, the essential TF [33,47]. One prominent approach of this can be seen in efforts to drug the BAF (aka SWI/SNF) complex. This complex remodels nucleosomes at promoters and enhancers to facilitate accessibility and therefore TF binding at these sites. Examples of this include targeting the AR pathway in prostate cancer and the PAX3-FOXO1 fusion pathway in rhabdomyosarcoma through inhibition of the BAF complex [33,47]. The recent development of compounds targeting this complex appear highly promising, with several progressing to clinical trials [48].

While efforts to directly drug TFs themselves have long been attempted [46], they have often failed to deliver on their promise. A major exception to this are hormone receptor targeting compounds [49]. New promising chemical biology approaches provide a glimmer of optimism for targeting these previously undruggable or intractable proteins.

*MYC* is among the most commonly deregulated genes in cancer, however, the MYC protein has been deemed “undruggable” due to intrinsically disordered regions in its structure [50]. The challenges associated with small-molecule inhibition of MYC have caused research efforts to develop approaches to disrupt MYC's transcriptional activity through avenues other than its DNA binding domain, such as by targeting stabilising posttranslational modifications of MYC. Unfortunately, these approaches have had limited success in clinical trials.

OMO-103, a recombinant “miniprotein” based on the structure of Omomyc, is the first MYC inhibitor to progress to clinical trials with promising stage 1 results so far [51]. MYC normally forms a heterodimer with its binding partner MAX via its basic helix-loop-helix domain. This heterodimerisation results in conformational changes which allow MYC/MAX to bind DNA. Omomyc resembles the structure of the wild-type MYC protein, with just four amino acid alterations in the leucine zipper [52,53]. The resulting protein gained the ability to homodimerise, and can heterodimerise with both wild-type MYC and MAX. Once dimerised, Omomyc inhibits DNA binding, thereby functioning as a dominant negative MYC inhibitor.

Nano and monobody based approaches have also been adapted to target TFs and their pathways. These have the benefit of being cell permeable [54] and can specifically target certain epitopes. In particular, these approaches have been used to develop compounds to target the TF STAT3. SBT-100 and MS3-6 bind to STAT3 and prevent it from binding to DNA [55,56].

## Targeted protein degradation approaches to degrade transcription factors

One of the most exciting recent advances in chemical biology is the growing field of targeted protein degradation (TPD) strategies. Such strategies employ proteolysis targeting chimeras (PROTACs) to exploit the cells own protein-degradation system by specifically directing proteins to the proteasome [57]. PROTACs are heterotrimeric, bifunctional molecules which consist of a linker region conjugated to an E3 ligase ligand at one end, and a ligand specific for the protein of interest at the other. PROTACs target the protein of interest for degradation via the proteasomal pathway by inducing proximity with an E3 ubiquitin ligase enzyme. Not only do PROTACs serve as a useful biochemical investigation tool *in vitro*, they have also proved to be promising therapeutic strategies for cancer patients, with a number of PROTAC-based drugs progressing to clinical trials [58]. ARV-471, a degrader compound targeting the ER hormone receptor TF, displayed promising results in its first in-human trial [59]. ARV-471 was well tolerated by breast cancer patients, and displayed potent ER degradation between 67 and 89 %, resulting in antitumour activity, supporting its progression to the ongoing phase III VERITAC-2 trial [60]. This highlights the potency of PROTAC technologies in the clinic, although compound and target specific variation in efficacy is to be expected as with any new class of drug.

**Figure 3:**
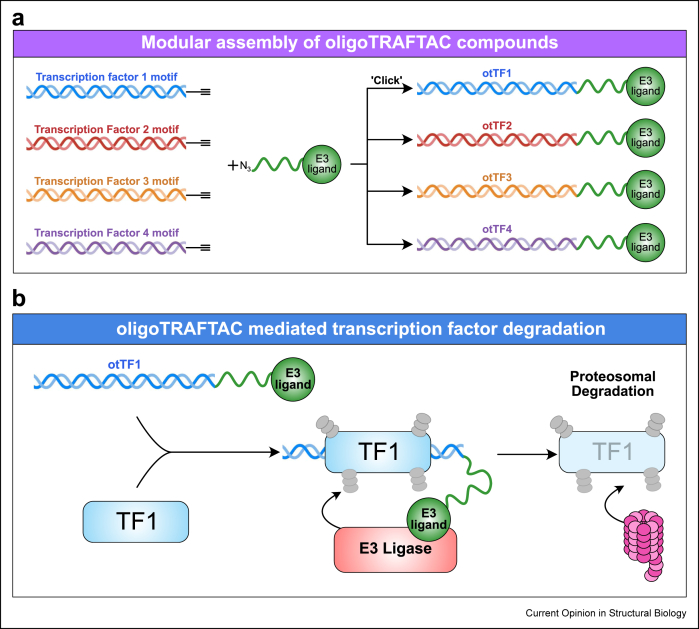
**Emerging approaches to drugtranscription factors**. **a)** Assembly of oligonucleotide-based PROTAC compounds to target transcription factors. Double-stranded oligonucleotides featuring transcription factor motifs can be used as modular ligands and linked to E3 ligase ligands via click chemistry. This allows for development of compounds to specifically target distinct transcription factors. **b)** Mechanism of action of oligoTRAFTAC compounds. Lineage-specific transcription factors bind to the synthetic double-stranded DNA oligonucleotide containing their specific binding motif. The E3 ligand portion of the oligoTRAFTAC recruits an E3 ligase to promote poly-ubiquitination and subsequent proteasomal degradation of the target transcription factor. Abbreviations: ot - oligoTRAFTAC; TF - Transcription factor.

For most intractable TFs, PROTAC design has not yet been possible due to challenges in ligand design. To overcome this, PROTAC technology has been adapted to harness the sequence-specific binding nature of TFs. The resulting molecules are oligonucleotide-based TF-targeting chimeras (oligoTRAFTACs and O’PROTACs) that share the heterotrimeric structure of PROTACs, using a short double-stranded oligonucleotide containing the binding motif specific for the TF of interest as a ligand connected to an E3 ligase ligand via a flexible linker region (Figure 3a) [61,62]. This motif-bearing oligonucleotide acts as a ligand for the TF and brings it into close proximity to an E3 ubiquitin ligase, directing it for ubiquitination and subsequent proteasomal degradation (Figure 3b). OligoTRAFTACs targeting MYC and Brachyury have shown strong target degradation *in vitro* and in a zebrafish *in vivo* model [61]. Similarly, co-degradation of Nrf2-sMaf, a TF heterodimer aberrantly overexpressed in cancer, was achieved by synthesising an oligonucleotide containing the specific antioxidant-response element recognised by Nrf2 [63]. While there are significant limitations to this current method, further improvements in compound stability [64] and delivery methods have the potential to yield a modular system that can be applied to any TF with a known DNA-binding motif (Figure 3a).

An alternative strategy recently developed for TPD leveraging TF motif specificity involves engineering artificial long noncoding RNA molecules (alnRNAs) [65]. alnRNA structures include an RNA aptamer capable of binding the TF of interest, and E3 ligase-recruiting sequences derived from the HOTAIR lncRNA. Similar to PROTACs and other TPD technologies, proximity is induced between the POI and the ubiquitinating enzyme, targeting it for proteasomal degradation. This approach has been effective for potent degradation of the oncogenic TFs MYC and NF-κB *in vitro* [65].

Another TPD strategy termed “molecular glue” degraders can target proteins of interest for degradation via similar mechanisms to PROTACs but offers advantages in terms of pharmacokinetics and drug-likeness [66,67]. Like PROTACs, molecular glues can induce protein–protein interactions; however, most molecular glues are monovalent unlike bivalent PROTAC compounds. For this reason, molecular glues are more challenging to design and the majority have thus far been discovered fortuitously. Recent chemoproteomics-based screens have resulted in the identification of potentially targetable residues on proteins, and the design of covalent ligands to react with these specific targets [68]. For example, the unique reactivity of cysteine has been exploited for the design of small molecule interactors. This has proved successful *in vitro* even for TFs commonly referred to as “undruggable”. Covalent ligand screening was used to discover that the EN4 ligand reacts with a specific cysteine residue within the predicted disordered region of MYC [69]. This interaction hinders the DNA-binding activity of MYC which in turn inhibits its transcriptional and tumourigenic activity. Similarly, covalent ligand-based molecular glues for NF-κB1 and CTNNB1 have been discovered which direct these two “undruggable” oncogenic TFs for proteasomal degradation [70,71]. Covalent chemoproteomics can also be applied to generate biochemical tools assessing the mechanisms of action of TFs. A cysteine-directed electrophilic compound was capable of binding the pioneer factor FOXA1, and redirecting its DNA-binding activity [72]. This compound altered FOXA1 binding preferences, remodelling activity and ultimately led to genomic relocalisation of FOXA1.

These outlined advances in ligand design, combined with the capability of hijacking cellular machinery and biochemical pathways, have led to the development of chemical inducer of proximities (CIPs) and transcriptional/epigenetic CIPs [73, 74, 75]. Using transcriptional/epigenetic CIPs, an interaction can be induced between two molecules, such as the transcriptional activator BRD4 and the BCL6 repressor TF. This leads to induced activation of pro-apoptotic BCL6 target genes through BRD4 recruitment [75]. Ultimately this causes the activation of cell death pathways, and the loss of viability of B-cell lymphoma cells. This example of the growing ability to generate ligands and manipulate the epigenome in a chemically tractable way is sure to lead to more advances and creative approaches to manipulate the transcriptional machinery for therapeutic benefit.

Overall, recent advances in the area of TPD are hugely promising for the potential clinical targeting of TFs. In particular, the ability to use a modular system such as oligoTRAFTAC and O’PROTAC to tailor the compound to a TF of choice may be a hugely beneficial advance in cancer treatment should it proceed to clinical stages.

## Conclusions and future perspectives

The recent advances in functional genomics, epigenomics, and chemical biology are facilitating a new drive to clinically target TFs. There are many obstacles remaining, including challenges in drug delivery for oligonucleotide-based therapies. However, there is enormous potential to tailor treatments to patients on the basis of the CRC rewiring in their cancer, instead of other prognostic biomarkers such as genotype.

Further limitations may include the selection of TFs to be targeted in specific cancers. Much of our knowledge of CRCs is built upon 2D *in vitro* cell line data [42]. Recent developments in epigenomic technology including transcriptomics, accessibility, and histone modification mapping at single-cell and spatial resolution will provide new avenues to map CRCs in more complex tissue types or in specific subpopulations of cells [76,77]. Such approaches can then be applied to 3D-model systems or primary tissues and biopsies to improve the resolution and accuracy of CRC mapping. These approaches could be used to unravel the TFs driving CRC in relapse populations of a tumour for instance [35]. In a similar vein, functional genomics screening approaches have moved beyond the limitations of 2D systems and are now widely applied in 3D models and also *in vivo* mouse studies [78,79]. These developments will improve the basis for targeting one TF over another, or more likely combinations of TFs, in specific cancers.

Future opportunities in TF drug design could come from recent technological advances that facilitate ligand identification for intrinsically disordered regions, which are a common feature of TFs [80]. Furthermore, Google DeepMind have recently released AlphaProteo, an AI system for the design of protein binders, trained on Protein Data Bank data and AlphaFold predicted structures [81]. AI may accelerate the process of drugging difficult to target proteins with strong potential for use as biochemical tools as well as therapeutics. These approaches could lead to the future generations of TF targeting compounds with potential for improved biochemical properties for clinical use.

Overall, the promise of a modular system to target lineage-specific TFs for distinct cancer types is a hugely attractive one. This area should see rapid advances in both the genomics and chemical biology aspects over the next 5–10 years.

## Author contributions

M.D and E.C conceived the topic of the review. M.D and M.B prepared figures. M.D and E.C wrote the manuscript. M.D, M.B and E.C approved the final version.

## Funding

The work of the Conway laboratory is supported by the Science Foundation Ireland/Irish Research Council Pathway program grant (21/PATH-S/9384) and Worldwide Cancer Research Grant (23-0085). E.C is supported by a Wellcome Trust early career award [225152/Z/22/Z]. M.B is supported by a University College Dublin, School of Biomolecular and Biomedical Sciences Research Scholarship.

## Declaration of competing interest

The authors declare that they have no known competing financial interests or personal relationships that could have appeared to influence the work reported in this paper.

## Data Availability

Data used in this article was generated by the Broad Institute and DepMap consortium and reanalysed and graphed for the purposes of this review [37, 40, 42].
